# Endovascular treatment modalities for basilar artery fenestration aneurysms: experience of two centers and literature review

**DOI:** 10.3906/sag-2006-352

**Published:** 2021-06-28

**Authors:** Mehmet KORKMAZ, Celal ÇİNAR, Ömer Fatih NAS, Bahattin HAKYEMEZ, İsmail ORAN

**Affiliations:** 1 Department of Radiology, Faculty of Medicine, Kütahya Health Sciences University, Kütahya Turkey; 2 Department of Radiology, Faculty of Medicine, Ege University, İzmir Turkey; 3 Department of Radiology, Faculty of Medicine, Bursa Uludağ University, Bursa Turkey

**Keywords:** Basilar artery fenestration, aneurysm, endovascular treatment

## Abstract

**Background/aim:**

The aim of this study is to present our experience on various endovascular treatment modalities of basilar fenestration aneurysms and reviewing our findings together with literature data.

**Materials and methods:**

A total of 26 basilar artery fenestration (BAF) aneurysms in 24 patients were endovascularly treated in two different neurointerventional radiology clinics. All patients had been evaluated with computed tomography (CT), CT angiography, and digital subtraction angiography before the procedure.

**Results:**

Aneurysms of all patients were successfully occluded (technical success rate 100%). Procedure-related complications were seen in two patients. Our comprehensive literature research revealed that studies related with the topic are composed mostly of case reports. In the literature, a total of 113 BAF aneurysms of 101 patients had been treated endovascularly in 46 studies between 1993 and 2019. Success rate of the treatment was reported as 97%, clinical success rate as 91%, and complication rate as 8.8%, while these rates are 100%, 92%, and 7.6% in our study, respectively.

**Conclusion:**

Although the number of cases is low in our study, to our knowledge, it is the widest series in the literature until today. Our results demonstrate that BAF aneurysms can be treated successfully and safely with different endovascular techniques, with more stent use in recent years.

## 1. Introduction

Basilar artery fenestration (BAF) is a rare vascular variation and its incidence is reported as 0.6–2.33% depending on the diagnostic modality chosen [1–3]. Fenestrations on this region increase tendency to aneurysm generation [2]. Complex geometric structure of the fenestration, proximity to lower cranial nerves, presence of sort of vital arterial structures, and deep and challenging nature of this region for surgery increase mortality and morbidity of surgical treatment in these aneurysms [4]. Endovascular treatment is becoming the first choice at the present time [5]. However, experiences on endovascular treatment are limited in the literature because of the rarity of these aneurysms. 

Our aim in this study is to present our experience on various endovascular treatment modalities of basilar fenestration aneurysms and reviewing our findings together with literature data.

## 2. Materials and methods

A total of 26 BAF aneurysms in 24 patients were endovascularly treated in two different neurointerventional radiology clinics. All patients had been evaluated with computed tomography (CT), CT angiography (CTA), and digital subtraction angiography (DSA) before the procedure. Ruptured and nonruptured aneurysms were included in the study. Patients with aneurysms unrelated to BAF were excluded. Aneurysms were divided into two groups: narrow-necked (neck diameter lower than 4 mm or dome/neck ratio higher than 2) and wide-necked (neck diameter higher than 4 mm or dome/neck ratio lower than 2) [6].

All patients were treated with endovascular method under general anesthesia after hospitalization. Systemic heparinization was performed in all patients ensuring an activated clotting time (ACT) of 250–300 s. After femoral artery puncture, angiograms of both carotid and vertebral arteries were obtained selectively. Endovascular protocols were divided into three groups according to type of aneurysms, presence of subarachnoid hemorrhage (SAH), clinical findings, and technical-material varieties at the time of the endovascular treatment. These groups are explained in the following subsections.

### 2.1. Standard coiling

It was used for narrow-necked aneurysms. Aneurysms were filled with different sized, controlled detachable coils (Target, Stryker, Fremont, CA, USA; GDC, Stryker, Fremont, CA, USA) after placing a microcatheter (Excelsior SL-10; Stryker; Natick, MA, USA) through a microguide (Radiofocus Guidewire GT; Terumo, Japan; Synchro; Stryker, Fremont, CA, USA; Traxcess; Microvention Terumo, Tustin, CA, USA) under fluoroscopic roadmap.

### 2.2. Balloon-assisted therapies (balloon modeling method)

These were used for wide-necked aneurysms. Nondetachable compliant balloons were used (Hyperform; Covidien, Irvine, CA, USA). Coiling was made after placing the balloon into the aneurysmatic branch or neighboring branch if it was a bifurcation aneurysm.

### 2.3. Stent-assisted therapies

These were used for wide-necked complex aneurysms. Stent-assisted treatment is generally avoided in patients with ruptured aneurysms, but it was used if avoidance was not possible. Dual antiplatelet therapy was given at least 4 h before the procedure with loading dose (300 mg acetylsalicylic acid and 600 mg clopidogrel) in ruptured aneurysms and 12 h with daily dose (300 mg acetylsalicylic acid and 600 mg clopidogrel) in unruptured aneurysms. Multiplate aggregometry test (Multiplate® analyzer, Roche Diagnostics; Switzerland) was made prior to the procedure in patients having loading dose. 

#### 2.3.1. Stent modeling method

In this method, coiling is made after placing a self-expendable stent (Solitaire; ev3, Inc., Irvine, California, USA) into the aneurysm neck under roadmap. 

#### 2.3.2. Flow diverter systems

##### 2.3.2.1. Telescopic stenting

In this method, self-expendable nested stents (LVIS Jr.; MicroVention, Aliso Viejo, CA, USA) are placed into aneurysmal neck if coiling is not appropriate. By this way flow diversion effect is ensured in the lumen of aneurysm.

##### 2.3.2.2. Flow-diverter stents (Pipeline embolization device; ev3-Covidien, Irvine, California, USA; Fred; MicroVention, Tustin, California, USA)

In this method, hardly braided flow-diverter stents are placed into the aneurysm neck and it is aimed to be treated by reducing blood flow inside. 

In our study, patients were followed with control angiograms made at early postoperative period and 6 months after the procedure. Antiplateled therapy was continued for 6 months in patients having stents. Angiographic treatment results were evaluated by using modified Raymond-Roy occlusion classification (mRROC) scale (class 1: complete occlusion, class 2: residual neck, and class 3: residual aneurysm; class 3a: contrast opacification within the coil interstices of a residual aneurysm and class 3b: contrast opacification outside the coil interstices). Furthermore, all patients were clinically followed for 6 months and modified Rankin scale (mRS) was used for clinical follow-up. mRS 0–2 were accepted as good clinical results.

## 3. Results

A total of 26 aneurysms of 24 patients were treated in two different facilities (16 patients in one center and 8 patients in another center) between 2000 and 2019. Eighteen of cases were women (18/24; 75%), 6 were men (6/24; 25%), and mean patient age was 56 (age range 40–76 years). There was SAH in 16 (16/24; 67%) and chronic headache in 6 patients (6/24; 25%). One patient (1/24; 4%) had respiratory depression due to aneurysmal compression and 1 patient (1/24; 4%) had incidentally detected BAF aneurysm. Two patients had 2 aneurysms for each, related with the fenestration. All of aneurysms were located on the proximal part of the basilar artery.

**Figure 1:**
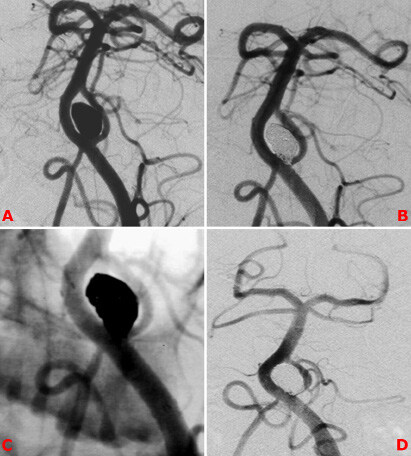
Fifty-two-year-old female patient presented with SAH. A narrow-necked proximal basilar artery aneurysm related to BAF is seen on DSA (A). It was treated with stent-assisted coiling (B, C). DSA control after 6 months (D).

**Figure 2:**
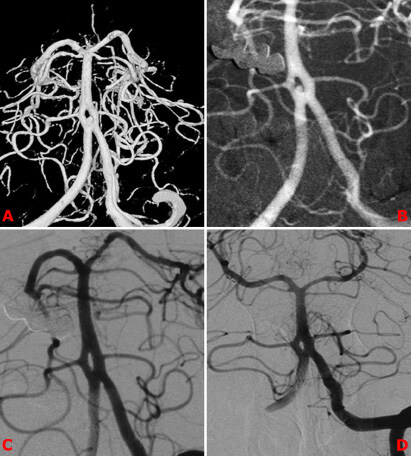
Sixty-year-old female patient presented with SAH. BAF related aneurysm is seen on 3D DSA images (A, B). It was treated with telescopic stenting-a homemade flow diverter technique (C). DSA control after 6 months (D).

**Figure 3:**
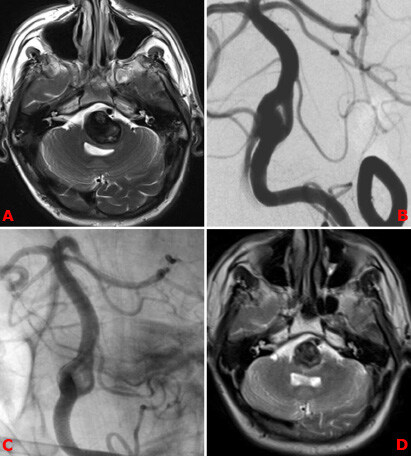
Fifty-year-old male patient presented with respiratory depression. A complex proximal basilar artery aneurysm related with BAF compressing the pons is seen on magnetic resonance imaging (MRI) (A) and DSA (B). It was treated with flow diverter stenting (C). MRI control after 1 month. Pons compression was regressed (D).

**Figure 4:**
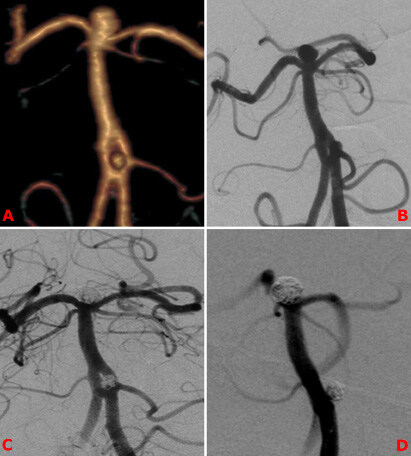
Sixty-two-year-old male patient presented with SAH. CTA and DSA show proximal BAF and relevant aneurysm. A basilar tip aneurysm is also seen (A, B). Both aneurysms have been treated with standard coiling (C). DSA control after 6 months (D).

Ten of aneurysms were narrow-necked and 16 were wide-necked generally. The mean diameter of aneurysms was 8 mm (range 2–30 mm). Embolization was made with standard coiling in 7, stent modeling in 8, telescopic stenting in 4, flow diverter stenting in 5, and balloon modeling in 2 patients (Figures 1–4). Aneurysms of all patients were successfully occluded (technical success rate 100%). Procedure-related complications were seen in two patients. One of these was brainstem perforating artery occlusion and the patient died because of brainstem infarction at postoperative day 12. Vertebral artery dissection of V3 segment happened during the procedure in the other case and it was treated with endovascular stenting at the same session. No additional neurologic complication was observed in the patient after the procedure and the follow-up period. The mean follow-up period was 62 months (range 1–120 months). Recurrence was not seen on radiologic follow-up. There was no neurologic impairment except one patient who died because of ischemic stroke (mRS scale 0–1). 

## 4. Discussion 

Fenestration is a rare anomaly of intracranial arteries and is related with embryogenesis. Fetal longitudinal neural arteries (FLNA) course along two sides of the hindbrain on its embryonic development and converge at week 5 on the midline forming basilar artery (BA). Fenestration can happen in any segment of BA where fusion of medial parts of FLNAs is defective. BAF is more likely to be proximally located, but rarely it can be located on the middle or distal parts of the artery [7]. All aneurysms in this study were proximally located.

While histopathologic examinations reveal normal structure of the lateral vessel wall, defects are seen in the medial wall of both edges of the fenestration and tunica media is seen to be absent on specimens. Furthermore, subendothelial lining has a different structure in the proximal segment and vessel wall does not contain elastin fibers, just like bifurcations. Structural weakness on the edge of a fenestration and hemodynamic effects of bifurcation increase the tendency of aneurysm formation, just like the case in intracranial bifurcations [3,5,8]. In this manner, recent studies reported aneurysm formation in 7% of cases with fenestrations and most of these have been shown to be on the vertebrobasilar junction [4].

Proximity to lower cranial nerves and numerous perforating arteries feeding the brainstem, insufficient surgical angle of view and complex geometric structure of the fenestration-related aneurysms are some of the factors making surgical treatment challenging. Kampos et al. [9] reported in their study that 20 BAF aneurysms were surgically treated, of which 14 were totally (70%) and 3 were subtotally (15%) closed. Transient lower cranial nerve paralysis was seen in 13 (65%) patients and permanent neurologic defect in 1 (5%) patient. One patient had died (5%). Otherwise, series with surgical treatment are limited in the literature. Although there are reports of successfully treated cases, surgical treatment is not the treatment of choice at the present day, because of factors like complex anatomy of this region, high procedural risks, and complexity of surgical techniques. Endovascular treatment has become the first-choice alternative to surgery in cerebral artery aneurysms, especially for the posterior circulation. Advances in endovascular techniques make successful treatment options possible for BAF aneurysms, as well as other types of aneurysms.

Our comprehensive literature research revealed that studies related with the topic are composed mostly of case reports. In the literature, a total of 113 BAF aneurysms of 101 patients had been treated endovascularly in 46 studies between 1993 and 2019 [4–8,10–26]. Success rate of the treatment was reported as 97%, clinical success rate as 91%, and complication rate as 8.8%. The second wider series after our study was reported by Zhu et al. [15] and their endovascular treatment success rate was 83%, clinical success rate 100%, and complication rate 0%, while these rates are 100%, 92%, and 7.6% in our study, respectively. These results are close to each other (Table 1).

**Table 1:** Comparison of the literature data, study of Zhu et al., and our study.

Studies	Number of aneurysms	Technical success rate	Complication rate	Clinical success rate
Our study	26	100%	7.6%	92%
Zhu et al. (2017)	12	83%	0%	100%
Literature data(1993–2020)	113	97%	8.8%	91%

Endovascular treatments in the literature are mostly standard coiling (63%) and stent-assisted techniques (25%). In our study, while stent-assisting composed the majority of therapies (65%), standard coiling (27%) was the second. Technical success, clinical success, and complication rates of the most commonly used modalities are similar (Table 2).

**Table 2:** Comparison of principle endovascular techniques used in the literature and our study.

Endovascular treatment techniques		Standard coiling	Parent artery Occlusion	Balloon-assisted TechniquesStent-assisted coiling	Stent-assisted techniques			Total
					Flow diverter systems			
					Telescopic stenting	Flow diverter stents		
Number of aneurysms	Our study	7(27%)	0 (0%)	2 (8%)	8(31%)	4(15%)	5 (19%)	26
	Literature	71(63%)	4 (3%)	10 (9%)	22 (19%)	3 (3%)	3 (3%)	113
Technical success rate	Our study	100%(7/7)	-	100%(2/2)	100%(8/8)	% 100(4/4)	100%(5/5)	100%
	Literature	97%(69/71)	100%(4/4)	90%(9/10)	100%(22/22)	100%(3/3)	100% (3/3)	97%
Complication rate	Our study	14% (1/7)	-	0% (0/2)	0% (0/8)	0% (0/4)	20%(1/5)	7.6%
	Literature	8%(6/71)	25%(1/4)	10%(1/10)	5%(1/22)	0%(0/3)	33% (1/3)	8.8%
Clinical success rate	Our study	86%(6/7)	-	100%(2/2)	100%(8/8)	100%(4/4)	80%(4/5)	92%
	Literature	91%(65/71)	75%(3/4)	90%(9/10)	95%(21/22)	100%(3/3)	67% (2/3)	91%

The literature and data from our study were evaluated together in 3 different periods according to endovascular materials used for neuroendovascular treatments: the first period, 1993–2001 (only coils were in use); the second period, 2002–2009 (balloon modeling technique was in use), and the third period, 2010–2019 (stents were in use). It was seen that most of the treatments were composed of standard coiling (18 aneurysms, 90%) in the first period, standard coiling (39 aneurysms, 85%) in the second period, and stent-assisted techniques (45 aneurysms, 62%) in the third period (Table 3).

**Table 3:** Different endovascular treatment modalities according to years together with our study data.

Years	Standard coiling	Parent artery occlusion	Balloon-assisted coiling	Stent-assisted techniques			Total
				Stent-assisted coiling	Flow diverters		
					Telescopic stenting	Flow diverter stents	
1993–2001	18 (90%)	2 (10%)	0	0	0	0	20 (100%)
2002–2009	39(85%)	2(4%)	5 (11%)	0	0	0	46 (%100)
2010–2020	21 (28%)	0	7 (10%)	30 (41%)	7 (10%)	8 (11%)	73(%100)
Total	78(56%)	4(3%)	12(9%)	30 (21%)	7(5%)	8 (6%)	139(%100)

Although standard coiling is still extensively used, different procedures, especially stent-assisted techniques are used more widely after advances in technology (technique and materials). Standard coiling is usually not successful in wide-necked and complex aneurysm. Stent-assisted techniques give more successful results in these types of aneurysms. Meckel et al. [14] treated a few complex giant BAF aneurysms in their study with flow-diverter stenting successfully as was in our study.

Four aneurysms had been treated with parent artery occlusion, which was not used in our study. However, both fenestrated arteries should be preserved, because major branches like anterior inferior cerebellar artery and posterior inferior cerebellar artery or small perforating arteries not seen on angiograms can originate from these. For this reason, this is not a recommended method [5].

When compared with the literature data, successful results were obtained with different endovascular procedures performed in our study and complication rates are quite low. The success rate for aneurysm treatment is expected to increase with the advances in technique and instrumentation. However, superiority of these techniques to each other cannot be revealed clearly. Treatment strategy can change depending on patient age, presence of hemorrhage, neurologic status, accompanying diseases, type of aneurysm, and experience-knowledge of the operator.

In conclusion, although the number of cases is low in our study, to our knowledge, it is the widest series in the literature until today. Our results demonstrate that BAF aneurysms can be treated successfully and safely with different endovascular techniques.

## Ethical approval

This retrospective study was approved by local institutional ethics committee.

## Informed consent

Informed consent was obtained from all individual participants included in the study.
